# Psoriasis in teledermatology: analysis of the 2016‒2020 period in Santa Catarina^☆^

**DOI:** 10.1016/j.abd.2022.01.010

**Published:** 2022-11-02

**Authors:** Maria Laura Orlandi Demo, Daniel Holthausen Nunes, Chaiana Esmeraldino Mendes Marcon

**Affiliations:** a Faculty of Medicine, Universidade do Sul de Santa Catarina, Tubarão, SC, Brazil; b Service of Dermatology, Hospital Universitário Polyodoro Ernani de São Thiago, Universidade Federal de Santa Catarina, Florianópolis, SC, Brazil

**Keywords:** Dermatology, Psoriasis, Public health, Teledermatology, Telemedicine

## Abstract

**Background:**

Psoriasis is a skin disease that affects 1.3% of Brazilians. The use of teledermatology (TD) in the public health sector has democratized access to dermatological care.

**Objective:**

To analyze TD exams with suspected and/or diagnosed psoriasis performed between 2016‒2020 in the state of Santa Catarina (SC).

**Methods:**

Analytical cross-sectional observational study that used secondary data collected from the records of TD exams from the Telemedicine and Telehealth System (TTS) of SC. The associations were evaluated by the chi-square test and Student's*t* test. The significance level was set at 5% (p < 0.05).

**Results:**

During the period, 6,146 TD exams were related to psoriasis, 58% due to the diagnosis provided by the reporting dermatologist and 42% exclusively due to the suspected disease on the request of the examination. The male sex predominated among the diagnoses of dermatosis (51%; p < 0.001). Regarding risk classification, psoriasis diagnoses were predominantly yellow (58.7%; p < 0.001) or blue (39.7%; p < 0.001) risk, respectively indicating moderate to severe psoriasis (referral to tertiary care) and mild psoriasis (treatment in the primary health care [PHC] level). True positive tests, suspected by PHC and diagnosed with psoriasis through TD, were 34.1% (p < 0.001).

**Study limitations:**

The TD service is available only for the public health network and analysis for a limited period (five years).

**Conclusions:**

Psoriasis diagnoses performed by TD, when compared to other dermatoses, tend to receive treatment at the primary (p < 0.001) or tertiary (p < 0.001) health care level, reducing the number of referrals to the secondary care level.

## Introduction

Psoriasis occurs globally^1^, ^2^ and affects approximately 125 million people worldwide.^3^ In Western countries, it affects 1.5 to 2% of the population^1^ and it is estimated to affect 1.3% of Brazilians.^2^, ^4^ It is a common, non-contagious,^2^ immunoinflammatory, chronic, and recurrent disease with cutaneous and articular involvement,^5^ the diagnosis of which is mainly clinical.^2^, ^6^

Teledermatology (TD) is considered a modality of Dermatology that applies information and communication technologies to remotely diagnose, monitor, treat, prevent, research, and educate.^7^ In the last decade, there has been a rapid growth of TD, with the expansion of its use in different care environments.^8^ It has been observed that it is one of the most important and employed categories of telemedicine, providing an excellent alternative to in-person dermatological consultations, with the use of electronic tools, which facilitate the provision of health care between clients and providers.^7^

The visual nature of dermatological practice makes it well suited for telemedicine care,^9^, ^10^ therefore TD increases access to high-quality dermatological care while maintaining clinical accuracy and adequate cost-effectiveness.^8^, ^9^ Moreover, TD democratizes access to specialized medicine, as it reaches populations in remote areas or areas lacking dermatologists, reduces long waiting periods for consultations, increases the number of assessed patients, and allows a faster diagnosis and treatment.^7^ Therefore, in countries with poor health access, such as Brazil, the use of TD is relevant, mainly due to its efficiency and direct connection with primary care.

The state of Santa Catarina (SC) is a national reference in TD services, 100% of its territory is covered by this technology – which has been expanding since 2018 to the entire country, after the choice of TD in SC by the Ministry of Health as the model to be implemented at a national level.^11^ From 2005 to November 2019, more than 130,000 TD exams were performed in the state.^12^ The differential of TD in the state of Santa Catarina is the development of the service based not only on screening and diagnosis but also on the integration of processes, protocols, and adequate technology at the central coordination and in the 286 TD examination services.^13^ Between January 2014 and June 2018, 33,112 patients were no longer referred to other centers but received treatment at the primary care level with clinical management supported by TD, thus preventing long waiting periods for consultations, traveling to other locations – often distant ones – and reducing costs.^13^ Therefore, it has been verified that TD contributes to the resolution of dermatological cases in primary care,^13^ in addition to collaborating with the educational training of general practitioners at this level of health care.^10^

In dermatological consultations carried out through the Unified Health System (SUS, *Sistema Único de Saúde*) in Brazil, one of the most frequent care demands is for psoriasis – especially in the Southern Region,^14^ which demonstrates the importance of the public dermatology service for the diagnosis and treatment of this dermatosis. Thus, primary care physicians must be competent to identify this common skin condition, and patients must receive health care compatible with the severity of their disease. Hence, TD in the state of SC contributes to efficient management of this disease, by screening patients according to their risk classification, so that moderate and severe conditions are promptly referred, while milder cases are treated at the Basic Health Unit (BHU), with clinical recommendations being provided by the dermatologist issuing the exam report.^15^

The aim of the present study was to analyze the TD exams with suspicion and/or diagnosis of psoriasis performed between the years 2016 and 2020 in the state of SC.

## Methods

### Study type

This is an observational, cross-sectional analytical study, with secondary data being collected from the records of TD exams from the Telemedicine and Telehealth System (TTS) of the state of SC.

### Study population

The study population comprises the TD exams in the state of SC carried out through the TTS, between January 2016 and December 2020, which had psoriasis as a diagnostic hypothesis in the exam request and/or psoriasis diagnosis in the report.

## Context and operability

The state of SC is divided into seven macroregions, which were considered by the study aiming to assess regional differences. They are Itajaí River Mouth, Great Florianópolis, Great Western, Midwestern and Santa Catarina Mountain Range, Northern/Northeastern Plateau, Southern, and Itajaí valley.

The TTS TD service follows standardized protocols and processes for requesting exams and issuing reports,^13^ both described in manuals provided to system users. Only registered BHU physicians and healthcare professionals can submit examination requests.^15^, ^16^

It encompasses an asynchronous (store-and-forward) system. Thus, the basic flow of dermatology telediagnosis in SC follows some basic steps:^15^, ^16^, ^17^ the patient has an appointment with a physician from the BHU who requests the dermatological exam, then a professional from the BHU – usually a nurse – performs the exam and sends the images captured with a camera and dermatoscope, of up to three lesions, to the TTS electronic site. A dermatologist then analyzes the case and issues the report (with the risk classification and guidelines for clinical management). The information generated by the physician who issued the report can be viewed by the requesting physician and by the patient on the TTS site. The examination request also contains the diagnostic suspicion of the BHU physician, which includes four possibilities: skin cancer, leprosy, psoriasis and other dermatoses. As of the year 2020, the diagnostic hypothesis options have been restricted to two: skin cancer and other dermatoses.^15^, ^16^

The risk classification used in the report includes five categories, which are defined according to the condition severity and support the clinical management: White - no need for intervention or follow-up; Blue - treatment at the BHU (according to the suggested dermatological conduct); Green - clinical-surgical evaluation with a specialist; Yellow – priority cases (refer to tertiary care); and Red – acute cases (refer to the Emergency Unit).^18^

## Data collection and analysis

Data, owned by the TTS and with restricted access, were collected through the Saiku-BI platform (open source license), which allows consultation of the records of TD exams. The database was created using Microsoft Office Excel software and the statistical analysis was performed using IBM SPSS Statistics software, version 20.0. Quantitative variables were described as measures of central tendency and dispersion, whereas qualitative variables were described as absolute (n) and relative (%) frequencies.

Tests that showed suspected and diagnosis of psoriasis were considered true positive ones, with the suspicion being provided by primary care when requesting the exam, and the diagnosis attributed by TD in the report. Thus, this parameter was verified directly on the Saiku-BI platform, using its cross-filtering of data.

The associations were evaluated using the chi-square test and Student's *t* test and the significance level was established at 5% (p < 0.05).

### Ethical aspects

The research project was approved by the Research Ethics Committee of Universidade do Sul de Santa Catarina – UNISUL (Counsel N. 4,283,737) and the state of Santa Catarina Health Secretariat – ​​SES/SC (Counsel N. 4,312,746), after waiver of the Free and Informed Consent Form (FICF).

## Results

During the period from 2016 to 2020, 141,657 TD exams were requested in SC. Of these, 4.3% were related to psoriasis, that is, 6,146 exams, with 58% (n = 3,566) due to the diagnosis provided by the dermatologist who issued the report and 42% (n = 2,580) due to the suspected disease when requesting the exam, which is determined by the BHU physician.

Males predominated among patients diagnosed with psoriasis (51%; n = 1,820), while females were the majority among suspected cases without a diagnosis of this dermatosis (54.8%; n = 1,415) and in the total number of exams (51.4%; n = 3,161); p < 0.001.

The mean age of patients was 46.9 ± 17.9 years. However, patients diagnosed with psoriasis had an older mean age (48.1 ± 16.6), when compared with cases suspected of psoriasis (45.3 ± 19.4); p < 0.001. When analyzing the age groups, differences were also observed: psoriasis diagnoses predominated between 31 to 49 years (31.4%; n = 1,118; p = 0.003) and between 50 to 60 (26%; n = 927; p < 0.001). The majority of suspected cases without a final diagnosis of psoriasis were in the age group ≤ 19 years (12.8%; n = 331; p < 0.001). There was no difference in the age groups of 20 to 30 and ≥ 61 years, with p = 0.906 and p = 0.887, respectively.

Phototype III predominate in the total of patients, with 42.3% of the exams (n = 2,599); p = 0.030.

When analyzing the seven macroregions of SC, it was observed that the Great Florianópolis macroregion had more exams related to psoriasis in the assessed period (30.2%; n = 1,859), followed by the Itajaí valley (17.6%; n = 1,084) and the Southern (14.1%; n = 866) macroregions. When comparing the diagnostic exams and those only suspected of psoriasis, Greater Florianópolis also had the highest number of diagnoses of this dermatosis (35.3%; n = 1,258; p < 0.001). On the other hand, suspected cases without a final diagnosis of psoriasis were the majority in the Itajaí River Mouth (5.6%; n = 144; p < 0.001), Northern/Northeastern Plateau (13.3%; n = 343; p = 0.037), Southern (16%; n = 414; p < 0.001) and Vale do Itajaí (20%; n = 516; p < 0.001) macroregions. There was no difference between the number of diagnoses and suspected cases between the Great Western (p = 0.514), Midwestern and Santa Catarina Mountain Range macroregions (p = 0.805).

Table 1 depicts the distribution of epidemiological characteristics.

**Table 1:** Epidemiological characteristics of patients with suspected and/or diagnosed psoriasis in TD exams in SC, 2016‒2020.

	Diagnosis of psoriasis	Suspected without confirmed of psoriasis	Total	p-value
	(n = 3566)	(n = 2580)	(n = 6146)	
**Sex**				**< 0.001**^a^
Female	49% (n = 1746)	54.8% (n = 1415)	51.4% (n = 3161)	
Male	51% (n = 1820)	45.2% (n = 1165)	48.6% (n = 2985)	
**Age (years)**				**< 0.001**^b^
Mean ± SD	48.1 ± 16.6	45.3 ± 19.4	46.9 ± 17.9	
**Age range**				**< 0.001**^a^
≤19 years	4.8% (n = 171)	12.8% (n = 331)	8.2% (n = 502)	< 0.001^a^
20‒30 years	12.8% (n = 457)	12.7% (n = 328)	12.8% (n = 785)	0.906^a^
31‒49 years	31.4% (n = 1118)	27.8% (n = 717)	29.9% (n = 1835)	0.003^a^
50‒60 years	26% (n = 927)	21.8% (n = 562)	24.2% (n = 1489)	< 0.001^a^
≥61 years	25% (n = 893)	24.9% (n = 642)	25% (n = 1535)	0.887^a^
**Phototype**				**0.030**^a^
I	5.2% (n = 184)	5.9% (n = 152)	5.5% (n = 336)	
II	20.3% (n = 724)	21% (n = 543)	20.6% (n = 1267)	
III	44.1% (n = 1572)	39.8% (n = 1027)	42.3% (n = 2599)	
IV	21.1% (n = 751)	22.7% (n = 585)	21.7% (n = 1336)	
V	7.8% (n = 277)	8.8% (n = 228)	8.2% (n = 505)	
VI	1.6% (n = 58)	1.7% (n = 45)	1.7% (n = 103)	
**Macroregion**				
Itajai River Mouth	2.1% (n = 75)	5.6% (n = 144)	3.6% (n = 219)	< 0.001^a^
Great Florianópolis	35.3% (n = 1258)	23.3% (n = 601)	30.2% (n = 1859)	< 0.001^a^
Great Western	9.8% (n = 348)	9.3% (n = 239)	9.6% (n = 587)	0.514^a^
Midwestern and Santa Catarina Mountain Range	12.7% (n = 454)	12.5% (n = 323)	12.6% (n = 777)	0.805^a^
Northern/Northeastern plateau	11.5% (n = 411)	13.3% (n = 343)	12.3% (n = 754)	0.037^a^
Southern	12.7% (n = 452)	16% (n = 414)	14.1% (n = 866)	< 0.001^a^
Itajaí valley	15.9% (n = 568)	20% (n = 516)	17.6% (n = 1084)	< 0.001^a^

a Pearson’s Chi-square.

b Student’s *t*.

The Body Mass Index (BMI) classification – Table 2 – was determined for adult patients (n = 5,645), excluding patients aged < 20 years due to the inadequacy of this type of classification for this age group. The distribution of underweight, overweight and the three degrees of obesity was similar between the groups. However, a healthy weight predominated among the suspected cases without a final diagnosis of psoriasis (29.9%; n = 673; p = 0.023).

**Table 2:** Body Mass Index (BMI) classification of patients aged ≥20 years with suspected and/or diagnosed psoriasis in TD exams in SC, 2016‒2020.

BMI classification	Diagnosis of psoriasis	Suspected without a final diagnosis of psoriasis	Total	p-value^a^
	(n = 3,394)	(n = 2,251)	(n = 5,645)	
Low weight	1.6% (n = 56)	1.6% (n = 37)	1.6% (n = 93)	0.986
Healthy weight	27.1% (n = 920)	29.9% (n = 673)	28.2% (n = 1593)	0.023
Overweight	35.6% (n = 1208)	36% (n = 811)	35.8% (n = 2019)	0.738
Obesity grade 1	21.9% (n = 743)	20.2% (n = 455)	21.2% (n = 1198)	0.131
Obesity grade 2	9.3% (n = 315)	8.2% (n = 185)	8.9% (n = 500)	0.169
Obesity grade 3	4.4% (n = 151)	4% (n = 90)	4.3% (n = 241)	0.412

a Pearson’s chi-square.

When requesting the TD exam, information regarding habits (smoking and alcohol consumption), comorbidities (diabetes mellitus - DM, systemic arterial hypertension - SAH, congestive heart failure - CHF and others) and infections (hepatitis B and C, human immunodeficiency virus – HIV and others) were also collected, as shown in Table 3. Absence of smoking and alcohol consumption were more frequent among suspected cases of psoriasis, with 62.7% (n = 1,617; p < 0.001) and 64 .5% (n = 1,665; p < 0.001) respectively. While among those diagnosed with psoriasis, there were more ex-smokers (24%; n = 855; p < 0.001), smokers (22.2%; n = 791; p < 0.001) and alcohol consumption: monthly (18.9%; n = 674; p < 0.001), 2 to 3 times/week (5.4%; n = 192; p = 0.045) and ≥ 4 times/week (3.7%; n = 132; p < 0.001). The distribution of comorbidities and infections was similar between the groups, except for DM, which was more frequently reported among psoriasis patients (10.3%; n = 367, p = 0.035).

**Table 3:** Habits, comorbidities and infections in patients with suspected and/or diagnosed psoriasis in TD exams in SC, 2016–2020.

	Diagnosis of psoriasis	Suspected without a diagnosis of psoriasis	Total	p-value^a^
	(n = 3,566)	(n = 2,580)	(n = 6,146)	
**Smoking**				
Never smoked	53.8% (n = 1920)	62.7% (n = 1617)	57.5% (n = 3537)	< 0.001
Ex-smoker	24% (n = 855)	19.6% (n = 505)	22.1% (n = 1360)	< 0.001
Smoker	22.2% (n = 791)	17.8% (n = 458)	20.3% (n = 1249)	< 0.001
**Alcohol consumption**				
Never	57.1% (n = 2035)	64.5% (n = 1665)	60.2% (n = 3700)	< 0.001
Monthly	18.9% (n = 674)	14.8% (n = 382)	17.2% (n = 1056)	< 0.001
2-4 times/month	14.9% (n = 532)	14.5% (n = 373)	14.7% (n = 905)	0.614
2-3 times/week	5.4% (n = 192)	4.3% (n = 110)	4.9% (n = 302)	0.045
≥4 times/week	3.7% (n = 132)	1.9% (n = 50)	3% (n = 182)	< 0.001
**Comorbidity**	**3**1.3% **(n = 1115)**	29.5% **(n = 762)**	**3**0.5% **(n = 1877)**	**0.146**
Dyslipidemia	10.5% (n = 373)	11.2% (n = 288)	10.8% (n = 661)	0.380
DM	10.3% (n = 367)	8.7% (n = 224)	9.6% (n = 591)	0.035
SAH	25.3% (n = 903)	24.1% (n = 623)	24.8% (n = 1526)	0.293
CHF	1.6% (n = 58)	1.4% (n = 37)	1.5% (n = 95)	0.546
**Other comorbidities**	8.5% **(n = 303)**	**8% (n = 207)**	8.3% **(n = 510)**	**0.506**
Infection	1.7% **(n = 62)**	**2% (n = 51)**	1.8% **(n = 113)**	**0.493**
Hepatitis B	0.7% (n = 25)	0.5% (n = 12)	0.6% (n = 37)	0.238
Hepatitis C	0.7% (n = 25)	0.8% (n = 20)	0.7% (n = 45)	0.737
HIV	0.4% (n = 16)	0.7% (n = 19)	0.6% (n = 35)	0.139
**Other infections**	0.5% **(n = 19)**	0.4% **(n = 11)**	0.5% **(n = 30)**	**0.555**

DM (Diabetes mellitus), SAH (systemic arterial hypertension) and HIV (Human immnunodeficiency virus).

a Pearson’s chi-square.

The number of exams related to psoriasis increased from 2016 with 19.2% of the total (n = 1,182) to 2019 (27.4%; n = 1,683), but it showed a sharp decreased in 2020 (7.2%; n = 444); p < 0.001. Most of the exams concomitantly evaluated two skin lesions (39.3%; n = 2,414; p < 0.001). Among the cases diagnosed with psoriasis, the diagnostic suspicion most frequently attributed by the BHU physician was psoriasis, in 58.7% of the cases (n = 2,093), but other dermatoses (39.2%; n = 1,399), skin cancer (1.7%; n = 62) and leprosy (0.4%; n = 13) were also suspected.

Psoriasis lesions were most frequent on the scalp (11.6%; n = 413; p = 0.035), posterior thorax (6.6%; n = 237; p = 0.002), abdomen (11.7%; n = 417; p < 0.001), posterior arm (14.6%; n = 521; p < 0.001), posterior forearm (23.2%; n = 828; p < 0.001), lumbar region (10.6%; n = 379; p < 0.001), anterior thigh (13.9%; n = 495; p < 0.001), posterior thigh (4.7%; n = 166; p < 0.001) and anterior leg (28.8%; n = 1,027; p < 0.001). In total, the most reported lesion distribution was localized in 76.9% of the exams (n = 4,725; p < 0.001). Patients with a final diagnosis of psoriasis had predominantly disseminated (23.4%; n = 833; p < 0.001) and generalized (3.6%; n = 129; p < 0.001), lesions, while the suspected cases had mostly single (11.1%; n = 286; p < 0.001) or localized lesions (80.2%; n = 2,068; p < 0.001).

Patients with a final diagnosis of psoriasis reported pruritus in 15.3% of the cases (n = 546; p < 0.001), a positive personal medical history in 3.4% (n = 120) and family history in 3.3% (n = 118); p < 0.001. Clinical information and the year of the TD exam request are shown in Table 4.

**Table 4:** Clinical information and year of TD exam request of patients with suspected and/or diagnosed psoriasis, SC, 2016‒2020.

	Diagnosis of psoriasis	Suspected without confirmed diagnosis of psoriasis	Total	p-value^a^
	(n = 3,566)	(n = 2,580)	(n = 6,146)	
**Year**				**< 0.001**
2016	17.3% (n = 618)	21.9% (n = 564)	19.2% (n = 1182)	
2017	21.1% (n = 753)	20% (n = 516)	20.6% (n = 1269)	
2018	23.7% (n = 845)	28% (n = 723)	25.5% (n = 1568)	
2019	25.5% (n = 910)	30% (n = 773)	27.4% (n = 1683)	
2020	12.3% (n = 440)	0.2% (n = 4)	7.2% (n = 444)	
**Number of sites with analyzed lesion**				**< 0.001**
One (1)	32.1% (n = 1143)	46.3% (n = 1194)	38% (n = 2337)	
Two (2)	40.4% (n = 1439)	37.8% (n = 975)	39.3% (n = 2414)	
Three (3)	27.6% (n = 984)	15.9% (n = 411)	22.7% (n = 1395)	
**Body location**				
Scalp	11.6% (n = 413)	9.9% (n = 255)	10.9% (n = 668)	0.035
Face	5.8% (n = 207)	8.4% (n = 217)	6.9% (n = 424)	< 0.001
Ear	3.8% (n = 137)	3.1% (n = 79)	3.5% (n = 216)	0.101
Anterior cervical region	0.5% (n = 18)	1.4% (n = 35)	0.9% (n = 53)	< 0.001
Posterior cervical region	5% (n = 178)	5.1% (n = 131)	5% (n = 309)	0.879
Anterior thorax	6.5% (n = 232)	5.9% (n = 153)	6.3% (n = 385)	0.358
Posterior thorax	6.6% (n = 237)	4.8% (n = 124)	5.9% (n = 361)	0.002
Abdomen	11.7% (n = 417)	7.1% (n = 182)	9.7% (n = 599)	< 0.001
Anterior arm	4.5% (n = 161)	6.1% (n = 157)	5.2% (n = 318)	0.006
Posterior arm	14.6% (n = 521)	9% (n = 231)	12.2% (n = 752)	< 0.001
Anterior forearm	7% (n = 249)	8.3% (n = 213)	7.5% (n = 462)	0.062
Posterior forearm	23.2% (n = 828)	15.5% (n = 400)	20% (n = 1228)	< 0.001
Dorsum of hand	5.7% (n = 203)	10% (n = 258)	7.5% (n = 461)	< 0.001
Hand (palm)	7.9% (n = 281)	10.6% (n = 274)	9% (n = 555)	< 0.001
Lumbar area	10.6% (n = 379)	4.6% (n = 118)	8.1% (n = 497)	< 0.001
Gluteal region	2.8% (n = 101)	2.6% (n = 66)	2.7% (n = 167)	0.514
Genital/Inguinal region	0.8% (n = 29)	0.8% (n = 21)	0.8% (n = 50)	0.998
Anterior thigh	13.9% (n = 495)	9.9% (n = 255)	12.2% (n = 750)	< 0.001
Posterior thigh	4.7% (n = 166)	2.9% (n = 75)	3.9% (n = 241)	< 0.001
Anterior leg	28.8% (n = 1027)	20.3% (n = 525)	25.3% (n = 1552)	< 0.001
Posterior leg	5.4% (n = 192)	5.2% (n = 133)	5.3% (n = 325)	0.692
Dorsum of foot	6.7% (n = 240)	12.1% (n = 313)	9% (n = 553)	< 0.001
Foot (plantar region)	4.6% (n = 163)	6.3% (n = 162)	5.3% (n = 325)	0.003
**Distribution of lesions**				
Single	5.4% (n = 191)	11.1% (n = 286)	7.8% (n = 477)	< 0.001
Localized	74.5% (n = 2657)	80.2% (n = 2068)	76.9% (n = 4725)	< 0.001
Disseminated	23.4% (n = 833)	11.5% (n = 296)	18.4% (n = 1129)	< 0.001
Generalized	3.6% (n = 129)	1.6% (n = 41)	2.8% (n = 170)	< 0.001

a Pearson’s chi-square.

Disease duration was reported in only 639 (17.9%) tests diagnosed with psoriasis. Of these, 45.7% (n = 292) had an evolution of one to five years, 20.7% (n = 132) of one to six months, 17.2% (n = 110) of six to ten years, 11.4% (n = 73) of 20 years or longer, 7.5% (n = 48) of 11 to 19 years, 7.2% (n = 46) of less than four weeks and 4.2% (n = 27) of seven to 11 months. It is noteworthy that the exam request could convey a different time of evolution for each assessed lesion; therefore, more than one temporal range was indicated in some exams.

Regarding the main risk classification (most severe on the exam), psoriasis diagnoses were predominantly yellow (58.7%; n = 2,095; p < 0.001) and blue (39.7%; n = 1,416; p < 0.001), which respectively indicate referral to an outpatient clinic for severe psoriasis and treatment at the BHU; of these, only 1.5% (n = 55) received a green classification. While the suspected cases, but without a final diagnosis of the dermatosis, were mostly green (72.6%; n = 1,873; p < 0.001), that is, referred to secondary care. Also among the suspected cases of psoriasis, 91 exams (3.5%; p < 0.001) were classified as white (no need for medical intervention) and three (0.1%; p = 0.042) as red – requiring immediate referral to the emergency unit. Most of the reports (95.1% of the total; n = 5,842) provided only one diagnosis/conclusion.

Psoriasis severity was defined as mild in 41.3% of reports (n = 1,472) and moderate to severe in 11.2% (n = 400), being undetermined in 48% (n = 1,711). Psoriasis was diagnosed in association with another dermatosis or diagnosis/conclusion in 4.9% (n = 173); in this case, with the majority also referred to general dermatology (1.3%; n = 45; p < 0.001) and in 0.2% (n = 8; p < 0.001) to phototherapy. Other skin diseases diagnosed concomitantly with psoriasis were, for example: chronic photodamage (0.4%; n = 14; p = 0.001), melanocytic nevus (0.3%; n = 11; p = 0.119), seborrheic dermatitis (0.3%; n = 10; p < 0.001), seborrheic keratosis (0.3%; n = 12; p = 0.650) and tinea (0.3%; n = 11; p < 0.001).

Regarding suspected cases without a final diagnosis of psoriasis, 62.8% (n = 1,619) were referred to general dermatology and 5.3% (n = 138) to pediatric dermatology. The most frequently diagnosed dermatoses in cases without confirmed psoriasis were: contact dermatitis (5.3%; n = 137; p < 0.001), xerosis cutis (4.7%; n = 121; p < 0.001), seborrheic dermatitis (3.4%; n = 89; p < 0.001), atopic dermatitis (3.2%; n = 83; p < 0.001), tinea (2.8%; n = 73; p < 0.001), chronic photodamage (1.1%; n = 29; p = 0.001), stasis eczema (0.9%; n = 22; p < 0.001), pityriasis versicolor (0.6%; n = 16; p < 0.001) and impetigo (0.5%; n = 14; p < 0.001).

Non-melanoma skin cancer, basal cell carcinoma (BCC) or squamous cell carcinoma (SCC), was diagnosed in 0.2% (n = 8) of patients with psoriasis and in 0.5% (n = 12) of patients with suspected psoriasis (p = 0.102). There was also a diagnosis of melanoma in a patient diagnosed with psoriasis (0.02%; n = 1; p = 0.395). The conclusions of the TD reports are shown in Table 5.

**Table 5:** Conclusion of the TD exam report in patients with suspected and/or diagnosed psoriasis, SC, 2016‒2020.

	Diagnosis of psoriasis	Suspected without a final diagnosis of psoriasis	Total	p-value^a^
	(n = 3,566)	(n = 2,580)	(n = 6,146)	
**Main risk classification**				
White	0% (n = 0)	3.5% (n = 91)	1.5% (n = 91)	< 0.001
Blue	39.7% (n = 1416)	19.1% (n = 493)	31.1% (n = 1909)	< 0.001
Green	1.5% (n = 55)	72.6% (n = 1873)	31.4% (n = 1928)	< 0.001
Yellow	58.7% (n = 2095)	4.7% (n = 120)	36% (n = 2215)	< 0.001
Red	0% (n = 0)	0.1% (n = 3)	0.05% (n = 3)	0.042
**Number of diagnoses**				**0.160**
One (1)	94.6% (n = 3374)	95.7% (n = 2468)	95.1% (n = 5842)	
Two (2)	5.1% (n = 183)	4.2% (n = 108)	4.7% (n = 291)	
Three (3)	0.3% (n = 9)	0.2% (n = 4)	0.2% (n = 13)	
**Diagnosis of psoriasis**	100% **(n = 3566)**	**0% (n = 0)**	58% **(n = 3566)**	**< 0.001**
Mild	41.3% (n = 1472)	0% (n = 0)	24% (n = 1472)	< 0.001
Moderate to severe	11.2% (n = 400)	0% (n = 0)	6.5% (n = 400)	< 0.001
Undefined severity	48% (n = 1711)	0% (n = 0)	27.8% (n = 1711)	< 0.001
**Diagnosis of psoriasis associated with another dermatosis/diagnosis**	4.9% **(n = 173)**	**0% (n = 0)**	2.8% **(n = 173)**	**< 0.001**

*Main risk classification: White – no need for intervention or follow-up; Blue - treatment at the BHU; Green – clinical-surgical evaluation with a specialist; Yellow – priority cases (refer to tertiary care); and Red – acute cases (refer to the Emergency Unit).

a Pearson’s chi-square.

The number of true positive tests was estimated, as shown in Table 6, by comparing the diagnostic hypothesis of the BHU physician *versus* the diagnosis of TD. From 2016 to 2020, 34.1% of the exams were true positive ones (n = 2,097; p < 0.001). The year 2017 reached the highest frequency regarding this parameter, with 41.4% (n = 526; p < 0.001); in turn, 2020 had the lowest frequency (1.6%; n = 7; p < 0.001). Great Florianópolis was the macroregion with the highest number of true positive exams (42.4%; n = 788; p < 0.001), while Itajaí River Mouth had the lowest (19.6%; n = 43; p < 0.001). Regarding the risk classification, there was a higher occurrence of yellow (59%; n = 1,307; p < 0.001) and blue cases (40%; n = 764; p < 0.001).

**Table 6:** True positive exams by year, macroregion and risk classification, SC, 2016‒2020.

	True positive exams	p-value^a^
**Year**		
2016	26.8% (n = 435)	0.030
2017	41.4% (n = 526)	< 0.001
2018	39.3% (n = 616)	< 0.001
2019	30.5% (n = 513)	< 0.001
2020	1.6% (n = 7)	< 0.001
Total in the period	34.1% (n = 2097)	< 0.001
**Macroregion**		
Itajai River Mouth	19.6% (n = 43)	< 0.001
Great Florianópolis	42.4% (n = 788)	< 0.001
Great Western	30.8% (n = 181)	0.078
Midwestern and Santa Catarina Mountain Range	35.1% (n = 273)	0.523
Northern/Northeastern plateau	27.2% (n = 205)	< 0.001
Southern	32% (n = 277)	0.153
Itajaí valley	30.4% (n = 330)	0.005
**Risk classification**		
White	0% (n = 0)	< 0.001
Blue	40% (n = 764)	< 0.001
Green	1.3% (n = 26)	< 0.001
Yellow	59% (n = 1307)	< 0.001
Red	0% (n = 0)	0.212

*Risk classification: White – no need for intervention or follow-up; Blue – treatment at the BHU; Green – clinical-surgical evaluation with a specialist; Yellow – priority cases (refer to tertiary care); and Red – acute cases (refer to the Emergency Unit).

a Pearson’s chi-square.

The clinical and epidemiological factors were compared among the seven macroregions of Santa Catarina in Table 7. It can be observed that the Northern/Northeastern Plateau region had more exams performed in male patients (52.5%; n = 396; p = 0.020), while the others macroregions showed no significant difference between the sexes. Regarding age, the youngest mean age was observed in the Great Western region (44.9 ± 19.1 years; p = 0.004) and the oldest in the Great Florianópolis macroregion (48.7 ± 16.9 years; p < 0.001).

**Table 7:** Macroregion according to clinical and epidemiological factors of TD exams in patients with suspected and/or diagnosed psoriasis, SC, 2016-2020.

	Itajai River Mouth (n = 219)	Great Florianópolis (n = 1859)	Great Western (n = 587)	Midwestern and Santa Catarina Mountain Range (n = 777)	Northern/Northeastern plateau (n = 754)	Southern (n = 866)	Itajaí valley (n = 1084)
**Sex**							
Female	49.3% (n = 108)	53% (n = 986)	48.6% (n = 285)	53% (n = 412)	47.5% (n = 358)	51.7% (n = 448)	52% (n = 564)
Male	50.7% (n = 111)	47% (n = 873)	51.4% (n = 302)	47% (n = 365)	52.5% (n = 396)	48.3% (n = 418)	48% (n = 520)
p-value^a^	0.523	0.097	0.142	0.342	0.020	0.849	0.664
**Age**							
Mean ± SD	46.5 ± 17.3	48.7 ± 16.9	44.9 ± 19.1	45 ± 18.7	48.1 ± 16.8	47.2 ± 18.4	45.4 ± 18.1
p-value^b^	0.706	< 0.001	0.004	0.002	0.050	0.564	0.002
**Risk classification**							
White	0.5% (n = 1; p = 0.201^a^)	1% (n = 19; p = 0.050^a^)	1.5% (n = 9; p = 0.912^a^)	0.8% (n = 6; p = 0.080^a^)	0.7% (n = 5; p = 0.047^a^)	1% (n = 9; p = 0.246^a^)	3.9% (n = 42; p < 0.001^a^)
Blue	18.7% (n = 41; p < 0.001^a^)	23.4% (n = 435; p < 0.001^a^)	42.8% (n = 251; p < 0.001^a^)	29.1% (n = 226; p = 0.203^a^)	31.6% (n = 238; p = 0.749^a^)	33.3% (n = 288; p = 0.132^a^)	39.7% (n = 430; p < 0.001^a^)
Green	55.7% (n = 122; p < 0.001^a^)	24.3% (n = 451; p < 0.001^a^)	30% (n = 176; p = 0.446^a^)	31.7% (n = 246; p = 0.852^a^)	35.7% (n = 269; p = 0.007^a^)	35.1% (n = 304; p = 0.011^a^)	33.2% (n = 360; p = 0.150^a^)
Yellow	25.1% (n = 55; p = 0.001^a^)	51.3% (n = 954; p < 0.001^a^)	25.6% (n = 150; p < 0.001^a^)	38.4% (n = 298; p = 0.151^a^)	32% (n = 241; p = 0.013^a^)	30.6% (n = 265; p < 0.001^a^)	23.2% (n = 252; p < 0.001^a^)
Red	0% (n = 0; p = 0.739^a^)	0% (n = 0; p = 0.254^a^)	0.2% (n = 1; p = 0.161^a^)	0.1% (n = 1; p = 0.281^a^)	0.1% (n = 1; p = 0.266^a^)	0% (n = 0; p = 0.483^a^)	0% (n = 0; p = 0.423^a^)
Diagnosis of psoriasis	**3**4.2% **(n = 75; p < 0.001**^a^**)**	67.7% **(n = 1258; p < 0.001**^a^**)**	**5**9.3% **(n = 348; p = 0.514**^a^**)**	**5**8.4% **(n = 454; p = 0.805**^a^**)**	**5**4.5% **(n = 411; p = 0.037**^a^**)**	52.2% **(n = 452; p < 0.001**^a^**)**	52.4% **(n = 568; p < 0.001**^a^**)**
Mild	10% (n = 22; p < 0.001^a^)	18.3% (n = 341; p < 0.001^a^)	35.8% (n = 210; p < 0.001^a^)	22.3% (n = 173; p = 0.239^a^)	24.9% (n = 188; p = 0.499^a^)	24.8% (n = 215; p = 0.514^a^)	29.8% (n = 323; p < 0.001^a^)
Moderate to severe	3.7% (n = 8; p = 0.081^a^)	7% (n = 130; p = 0.310*)	6.3% (n = 37; p = 0.832^a^)	6.9% (n = 54; p = 0.593^a^)	7.2% (n = 54; p = 0.437^a^)	6.8% (n = 59; p = 0.695^a^)	5.4% (n = 58; p = 0.089^a^)
Undefined severity	20.5% (n = 45; p = 0.014^a^)	42.5% (n = 791; p < 0.001^a^)	17.7% (n = 104; p < 0.001^a^)	29.3% (n = 228; p = 0.317^a^)	22.8% (n = 172; p = 0.001^a^)	20.8% (n = 180; p < 0.001^a^)	17.6% (n = 191; p < 0.001^a^)
Diagnosis of psoriasis associated with another diagnosis	3.7% **(n = 8; p = 0.445**^a^**)**	2.5% **(n = 47; p = 0.371**^a^**)**	4.3% **(n = 25; p = 0.026**^a^**)**	2.1% **(n = 16; p = 0.173**^a^**)**	4.4% **(n = 33; p = 0.006**^a^**)**	1.2% **(n = 10; p = 0.001**^a^**)**	3.1% **(n = 34; p = 0.480**^a^**)**

*Main risk classification: White – no need for intervention or follow-up; Blue – treatment at the BHU; Green – clinical-surgical evaluation with a specialist; Yellow – priority cases (refer to tertiary care); and Red – acute cases (refer to the Emergency Unit).

a Pearson’s chi-square.

b Student’s *t*.

The white risk classification was mainly observed in Itajaí Valley, comprising 3.9% of its exams (n = 42; p < 0.001). The blue risk classification was mainly observed in the Great Western region (42.8%; n = 251; p < 0.001), while Itajaí River Mouth was the macroregion with the most cases of green risk classifications (55.7%; n = 122; p < 0.001). The yellow risk was more frequent in Greater Florianópolis (51.3%; n = 954; p < 0.001), while the red risk classification occurred in only three macroregions: Great Western (0.2%; n = 1; p = 0.161), Midwestern and Santa Catarina Mountain Range (0.1%; n = 1; p = 0.281) and Northern/Northeastern Plateau (0.1%; n = 1; p = 0.266).

Regarding the severity of psoriasis, mild disease predominated in the Great Western (35.8%; n = 210; p < 0.001) and moderate to severe disease in the Northern/Northeastern Plateau (7.2%; n = 54; p = 0.437), while Greater Florianópolis had the majority of cases with undetermined severity (42.5%; n = 791; p < 0.001). The Northern/Northeastern Plateau also had the highest number of psoriasis diagnoses associated with another dermatosis in the TD report (4.4%; n = 33; p = 0.006).

## Discussion

Psoriasis has no predilection for either sex;^2^, ^19^, ^20^ however, more diagnoses of this dermatosis were made in males (51%; n = 1,820), although females predominated regarding the total number of TD exams in the period (51.4%; n = 3,161); p < 0.001. Perhaps this is due to the fact that there is a greater demand for health services by women,^21^ which results in more late diagnoses and complications of chronic health conditions in men.^22^ It is also observed that patients with undiagnosed active psoriasis tend to be male and have a lower level of schooling.^23^ Additionally, in Brazil, the prevalence of psoriasis is estimated to be different between the sexes:^24^ 1.15% of women and 1.47% of men.

Young adults are more often affected by psoriasis, which is rare in the pediatric population, and there is also a bimodal peak of disease onset, with differences reported in the literature:^20^ the first between 20 to 29 or 30 to 39 years and the second between 50 to 59 or 60 to 69 years. This age distribution is compatible with that of the present study since the mean age of patients diagnosed with psoriasis was 48.1 ± 16.6. Moreover, the 31 to 49 and 50 to 60 age groups accounted for 57.4% (n = 2,045) of psoriasis diagnoses, which were rare in patients aged ≤ 19 years (4.8%; n = 171).

The distribution of TD exams in terms of phototype, mostly in the lower types – I, II and III (68.4%; n = 4,202) – is consistent with the 2010 Brazilian census,^25^ in which the white skin color was self-declared by 84% of the population of the state of Santa Catarina. These phototypes also accounted for the majority of the diagnoses of psoriasis (69.6%; n = 2,480), a dermatosis that is more prevalent among Caucasian individuals.^26^

Between 2016 and 2020, the Great Florianópolis macroregion had the highest number of exams related to psoriasis (30.2%; n = 1,859), diagnoses of this dermatosis (35.3%; n = 1,258), and non-confirmed suspected cases (23, 3%; n = 601). This significantly higher number of exams can be explained by the extensive medical care available in the state capital, since, in 2020, Florianópolis had a ratio of 10.68 physicians for every 1,000 inhabitants, while the cities in the interior of SC had a 2.03 ratio.^27^ In Brazil, the concentration of physicians in large cities, coastal regions and regions with greater socioeconomic development is significant, being also higher in the private sector to the detriment of the SUS.^28^ It should also be noted that the TD center is located in Florianópolis; thus, physicians in the region tend to know more about the existence of the service and, for this reason, request more exams.

The pathogenic potential of the inflammatory mediators involved in psoriasis goes beyond the skin lesions and affects the body as a whole, which determines a state of systemic inflammation^29^ and the development of comorbidities. Obesity is highly incident and prevalent in patients with psoriasis, in addition to being related to greater disease severity;^24^ its prevalence is estimated to be 25.6%.^4^ Among the psoriasis diagnoses of the assessed period, concomitant obesity was observed in 35.6% (n = 1,209) of them. Individuals with psoriasis also had higher prevalence rates of smoking and alcohol consumption than the general population,^24^ a fact observed in the study when comparing the cases with a final diagnosis of psoriasis with those only suspected but not confirmed.

Comorbidities such as dyslipidemia, DM and SAH are also related to psoriasis, with prevalence rates estimated at 20.4%, 11.7% and 26.9%, respectively;^4^, ^24^ however, they were lower among the cases of psoriasis diagnosed in the present study: 10.5% (n = 373), 10.3% (n = 367) and 25.3% (n = 903). Therefore, it is necessary to encourage lifestyle changes (cessation of smoking and alcohol consumption, physical exercise and a balanced diet) in the clinical management of psoriasis, aiming to contribute to disease control.

HIV infection is a recognized risk factor for the development of psoriasis ^24^ and was found in 0.4% (n = 16) of diagnosed cases in the period. Moreover, it is important to pay attention to the presence of hepatitis in patients with psoriasis, as liver function can be worsened by hepatotoxic systemic treatments or reactivated infection by immunosuppressants, both with potentially severe outcomes.^24^ Hepatitis B was reported in 0.7% (n = 25) of psoriasis diagnoses, and hepatitis C in 0.7% (n = 25).

There is an important genetic predisposition in psoriasis^24^ and 30% of patients report an affected family member;^2^ however, only 3.3% (n = 118) of the patients diagnosed with psoriasis in the assessed period had a positive family history of morbidity. Likewise, pruritus, with an estimated prevalence of around 80%,^24^ was reported in only 15.3% (n = 546) of the diagnoses. This alerts to the possibility that the anamnesis data may be underestimated and are not filled out by the physicians requesting the TD exams, which jeopardizes the evaluation of the dermatological condition by the dermatologist that issues the report.

The body sites with the most lesions diagnosed as psoriasis in the assessed period – anterior leg (28.8%; n = 1,027), posterior forearm (23.2%; n = 828), posterior arm (14.6%; n = 521), anterior thigh (13.9%; n = 495), abdomen (11.7%; n = 417), scalp (11.6%; n = 413) and lumbar region (10.6%; n = 379) – were compatible with the literature: preference for the extensor region of the limbs, trunk, sacral region and scalp.^5^, ^6^ Regarding lesion distribution, it was described as localized by the BHU physician in 76.9% of the diagnoses of psoriasis, but it is inferred that this information is possibly wrong when compared to the severity of the diagnosed conditions since 58.7% (n = 2,095) received a yellow classification – indicating moderate to severe psoriasis, usually extensive, and requiring referral to a tertiary outpatient clinic.

Disease duration was another piece of information that was quite absent in the exam requests, reported in only 17.9% (n = 639) of the psoriasis diagnoses, but indicating that the diagnoses were late ones, after disease evolution that lasted years in a considerable number of individuals. This fact may indicate that patients delayed in seeking medical help or that the psoriasis conditions were being managed with difficulty in PHC. Currently, with the advent of TD, access to the public dermatology service has been facilitated and democratized in the state. Patients with psoriasis are often undiagnosed,^23^ undertreated, or even go untreated because the cutaneous involvement is the main and – often – the only manifestation of the disease,^29^ whose potential for systemic inflammation is still mistakenly underestimated by some medical professionals.

Before the implementation of TD, SC had a long waiting list for dermatological consultations in the SUS, from which patients with skin conditions ranging from benign to severe, with no definition of priority, were all referred to general dermatology outpatient clinics (secondary referral).^30^ TD established an effective screening and risk classification system,^30^ which allowed optimizing dermatologic referrals according to their level of priority,^13^ shortening the waiting time for diagnosis (from six months to 72 hours),^31^ and reducing the costs associated with in-person consultations.^32^ Thus, cases screened by TD in the white, blue, yellow and red categories do not require in-person evaluation by the secondary referral,^18^ being referred directly to other levels of health care or receiving treatment/care at the BHU.

In this sense, considering the TD exams related to psoriasis between 2016 and 2020, 68.6% (n = 4,218) of the exams prevented referral to secondary care. This percentage is higher than the 60.6% found in another study,^30^ which evaluated all exams performed by the TD of the TTS/SC in the period from 2015 to 2018.

When analyzing only the diagnoses of psoriasis, there is an even higher frequency of exams that did not require going through the general dermatology sector: 98.4% (n = 3,511). Of these, 58.7% (n = 2,095), categorized as yellow risk classification (moderate/severe psoriasis)^18^ were directly referred to tertiary care (severe psoriasis outpatient clinic). The respective TD report already contains recommendations for laboratory and imaging tests, to be done before the day of the consultation.^5^ It is the responsibility of the BHU physician to request them, aiming to anticipate the beginning of the systemic or biological treatment – when necessary. On the other hand, 39.7% (n = 1,416) of cases classified as blue risk (mild psoriasis)^18^ received treatment in PHC with the BHU physician, based on guidelines also provided in the examination report, according to the clinical management protocols of TD.^5^

Regarding the cases of suspected psoriasis but non confirmed, the evaluation of the secondary referral was prevented in only 27.4% (n = 707) of the exams. Cases classified as blue risk (such as actinic keratosis, seborrheic dermatitis and tinea) ^18^ and therefore treated at the BHU comprised 19.1% (n = 493). Cases classified as yellow risk and referred to tertiary care (for example, alopecia, collagenosis and leprosy),^18^ were 4.7% (n = 120), while whites (seborrheic keratosis, melanocytic nevus, chronic photodamage and other benign conditions),^18^ without the need for medical intervention, were 3.5% (n = 91). These received recommendations on their skin condition and the need for sun protection in the BHU. It is noteworthy the occurrence of 0.1% (n = 3) of cases classified as red risk (for example, acute cases of drug reaction, erythroderma, extensive vasculitis, or cellulitis of the face),^18^ which required immediate referral to the emergency department.

The diagnostic compatibility of TD *versus* in-person dermatological evaluation has shown to be quite high for inflammatory dermatoses such as psoriasis, reaching a diagnostic agreement rate of ≥ 90% in a study carried out in the city of São Paulo with an asynchronous TD model.^33^ On the other hand, the true positive tests (suspected by PHC and diagnosed with psoriasis during the evaluation of TD), totaled 34.1% – a value higher than the 19.02% obtained by a study that analyzed the diagnostic compatibility of PHC *versus* TD of the TTS/SC in skin cancer^32^ between January and July 2012. Significant differences were also found between the level of true positive tests in the state macroregions, with the highest levels found in Greater Florianópolis (42.4%) and the lowest in Itajaí River Mouth (19.6%) – perhaps due to better medical coverage in the state capital.^27^ It is noteworthy that the diagnostic assertiveness of PHC, as well as its effectiveness regarding psoriasis cases, tends to improve in the long term, as TD provides technical improvement to general practitioners at this level of health care.^10^

It should be noted that the year 2020 had the lowest number of exams in the period (n = 444) due to the COVID-19 pandemic, which restricted medical care not related to this infection, as well as TD exams. In addition to showing the lowest number of true positive tests in the period (1.6%), this year, the diagnostic hypotheses indicated by the requesting physician were restricted to only two: skin cancer and other dermatoses,^15^ aiming at facilitating exam requests.

The TTS is one of the largest telemedicine networks in the southern hemisphere and performs an average of 80,000 exams per month.^31^ Its TD service is a pioneering screening and teleconsultation model. Considering that dermatologists are heterogeneously distributed throughout the Brazilian territory, concentrating in more populous cities and with better Human Development Index (HDI),^34^ which is also a reality of the medical demography in SC,^27^ the TD service in Santa Catarina has shown to be a viable solution to meet the lack of specialized physicians in the public health network, especially in the most remote areas of the state.^32^

As a limitation of the present study, the analysis of TD service is available only in the public health network and for a limited period (five years).

## Conclusion

Suspected psoriasis exams in the PHC evaluation and with a diagnosis confirmed by TD, between the years 2016 and 2020 in SC, comprised 34.1% of the exams related to the dermatosis (p < 0.001), a percentage that can be considered low for a highly prevalent disease.

In the same period, TD was able to diagnose psoriasis in 3,566 patients and rule out the disease in 2,580 cases considered suspect in the clinical analysis of PHC. Thus, referral to secondary care was prevented in 98.4% of exams diagnosed with the disease, which referred cases of moderate/severe psoriasis directly to tertiary care (58.7%) and supported the treatment of mild cases at the BHU (39.7%). Psoriasis diagnoses performed by TD, when compared to other dermatoses, are more likely to receive treatment at the primary (p < 0.001) or tertiary (p < 0.001) levels of health care.

Therefore, it is concluded that TD in the state of Santa Catarina is efficient in the management of psoriasis cases treated via SUS, by decreasing the need for in-person dermatological assessments and increasing the effectiveness of its medical care in PHC.

## Financial support

None declared.

## Authors' contributions

Maria Laura Orlandi Demo: Design and planning of the study; collection of data; statistical analysis; interpretation of data; critical review of the literature; drafting and editing of the manuscript; approval of the final version of the manuscript.

Daniel Holthausen Nunes: Effective participation in research orientation; design and planning of the study; collection and interpretation of data; critical review of the manuscript; approval of the final version of the manuscript.

Chaiana Esmeraldino Mendes Marcon: Effective participation in research orientation; design and planning of the study; statistical analysis; interpretation of data; critical review of the manuscript; approval of the final version of the manuscript.

## Conflicts of interest

Maria Laura Orlandi Demo: none declared.

Daniel Holthausen Nunes: has the following conflicts of interest:

Speaker: Abbvie, Bayer, Janssen, Leo, Novartis and Pfizer;

Preparation of scientific material: Abbvie, Janssen, Novartis and Pfizer;

Financial support to participate in scientific events: Abbvie, Janssen, Leo, Novartis, Pfizer and Sanofi;

Advisory Board: Abbvie, Bayer, Janssen and Novartis.

Chaiana Esmeraldino Mendes Marcon: none declared.
